# Recovery of Metagenome-Assembled Genomes from a Human Fecal Sample with Pacific Biosciences High-Fidelity Sequencing

**DOI:** 10.1128/mra.00250-22

**Published:** 2022-05-09

**Authors:** Florian Plaza Oñate, Hugo Roume, Mathieu Almeida

**Affiliations:** a Université Paris-Saclay, INRAE, MGP, Jouy-en-Josas, France; University of Rochester School of Medicine and Dentistry

## Abstract

Here, we report the recovery of 89 metagenome-assembled genomes (MAGs) derived from a human fecal sample subjected to Pacific Biosciences (PacBio) high-fidelity (HiFi) sequencing. A total of 9 MAGs consisted of complete circular contigs, and 45 MAGs were high-quality draft genomes according to the minimum information about a metagenome-assembled genome (MIMAG) standards.

## ANNOUNCEMENT

Recent studies have shown that long-read sequencing technologies improve the contiguity of metagenomic assemblies and enable the recovery of repeated regions compared with short-read alternatives (1, 2). To assess long-read technologies and evaluate dedicated bioinformatics tools, we performed deep sequencing of a human fecal metagenome with the PacBio Sequel II system.

A fecal sample was self-collected by a 30-year-old healthy French volunteer for whom written consent was obtained. The sample was stored immediately in a stabilizing solution (RNAlater) following the International Human Microbiome Standards (IHMS) SOP_05_V2 (3, 4). DNA was extracted from 200 mg of fecal material following the IHMS SOP_07_V2 (3, 5). A total of 500 ng of high-molecular-weight DNA was used to build an unamplified nonmultiplexed library with the SMRTbell express template prep kit 2.0 (Pacific Biosciences) following manufacturer recommendations for metagenomics (6). Then, a 30-hour sequencing run was performed on a Sequel II device using Chemistry v2.0. Finally, removal of adapter sequences, read quality control, and generation of circular consensus sequencing (CCS) reads were performed through a dedicated pipeline (7).

**FIG 1:**
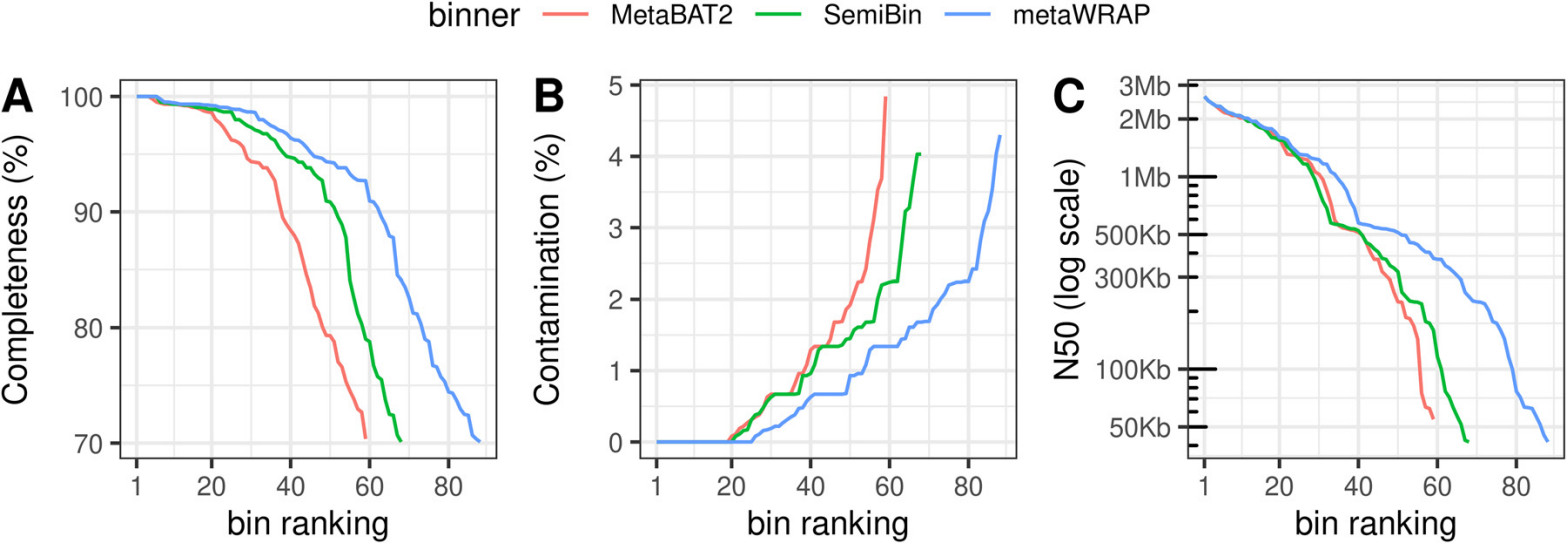
Rank order plots comparing the quality of MAGs produced by the three binning methods. Estimated completeness (A), estimated contamination (B), and *N*_50_ statistics (C) taken from the CheckM output report are shown. Only MAGs with completeness of ≥70% and contamination of ≤5% are considered.

Below, default parameters were used for all software unless otherwise specified. Reads shorter than 1,000 bp or aligned to the human genome (GenBank accession number GCA_009914755.3) with minimap v2.24 (8) (parameters, -x asm20) were discarded. In total, 1,645,079 reads with a median quality value of 40 were obtained for a cumulative length of 13,012,430,198 bp. The median length of the sequencing reads was 7,620 bp (Q1 = 5,936 bp; Q3 = 9,635 bp). Metagenomic assembly was performed with Flye v2.9 (9) (parameters: ‐‐pacbio-hifi ‐‐meta), and obtained contigs shorter than 2,500 bp were filtered out. The assembly consisted of 9,253 contigs (including 9 circular contigs of ≥1 Mb) with a cumulative length of 596,522,308 bp. *N*_50_ and *L*_50_ values were 164,736 bp and 628, respectively. Contig binning was performed with MetaBAT v2.12.1 (10) and SemiBin v0.5.0 (11) (parameters: ‐‐environment human_gut). Results from both tools were combined with the bin_refinement module implemented in metaWRAP v1.3.2 (12). Metagenome-assembled genome (MAG) quality was assessed with checkm v1.1.3 (13) except for one eukaryotic MAG for which BUSCO v5.2.2 (14) was used. A total of 89 MAGs with an estimated completeness of ≥70%, with a contamination of ≤5%, and passing the chimera detection implemented in GUNC v1.0.5 (15) were selected (Fig. 1). These MAGs were annotated subsequently with Prokka v1.14.5 (16), and taxonomic classification was performed with GTDB-Tk v1.5.0 (17). In total, 9 MAGs consisted of complete circular contigs, and 45 MAGs were high-quality draft sequences according to the minimum information about metagenome-assembled genome (MIMAG) standards (18). Notably, all 88 prokaryotic MAGs had at least 1 complete 16S rRNA gene. MAGs were mainly bacteria (87/89), 1 was an archaea (min17_bin38), and the largest (11.6 Mb) was an unicellular eukaryote of the genus *Blastocystis* (min17_eukbin1). We compared our prokaryotic MAGs with the representative genomes of the UHGG catalogue v2 (19) using fastANI v1.33 (20). Three MAGs corresponded to species not represented in the Unified Human Gastrointestinal Genome (UHGG) collection (average nucleotide identity cutoff = 95%). Remarkably, 30 MAGs had better assembly statistics than the UHGG representatives according to a composite score defined as *completeness* – (5 × *contamination*) + log(*N*_50_).

### Data availability.

Sequencing data (accession ERX7722845) and primary metagenome assembly (accession ERZ4963561) were deposited in the European Nucleotide Archive (ENA) under BioProject accession number PRJEB50473. Prokka annotation reports, MAG sequences, and related metadata were deposited in the INRAE data portal (data set S63W9S).

## References

[1] Bickhart DM, Kolmogorov M, Tseng E, Portik DM, Korobeynikov A, Tolstoganov I, Uritskiy G, Liachko I, Sullivan ST, Shin SB, Zorea A, Andreu VP, Panke-Buisse K, Medema MH, Mizrahi I, Pevzner PA, Smith TPL. 2022. Generating lineage-resolved, complete metagenome-assembled genomes from complex microbial communities. Nat Biotechnol doi:10.1038/s41587-021-01130-z. 34980911

[2] Gehrig JL, Portik DM, Driscoll MD, Jackson E, Chakraborty S, Gratalo D, Ashby M, Valladares R. 2022. Finding the right fit: evaluation of short-read and long-read sequencing approaches to maximize the utility of clinical microbiome data. Microb Genomics 8. doi:10.1099/mgen.0.000794. PMC9176275 35302439

[3] Costea PI, Zeller G, Sunagawa S, Pelletier E, Alberti A, Levenez F, Tramontano M, Driessen M, Hercog R, Jung F-E, Kultima JR, Hayward MR, Coelho LP, Allen-Vercoe E, Bertrand L, Blaut M, Brown JRM, Carton T, Cools-Portier S, Daigneault M, Derrien M, Druesne A, de Vos WM, Finlay BB, Flint HJ, Guarner F, Hattori M, Heilig H, Luna RA, van Hylckama Vlieg J, Junick J, Klymiuk I, Langella P, Le Chatelier E, Mai V, Manichanh C, Martin JC, Mery C, Morita H, O'Toole PW, Orvain C, Patil KR, Penders J, Persson S, Pons N, Popova M, Salonen A, Saulnier D, Scott KP, Singh B, et al. 2017. Towards standards for human fecal sample processing in metagenomic studies. Nat Biotechnol 35:1069–1076. doi:10.1038/nbt.3960. 28967887

[4] International Human Microbiome Standards (IHMS). Standard operating procedure for fecal samples preserved in stabilizing solution self-Collection, laboratory analysis handled within 24 hours to 7 days. INRAE, Jouy-en-Josas, France. http://www.human-microbiome.org/index.php?id=Sop&num=005.

[5] International Human Microbiome Standards (IHMS). Standard operating procedure for fecal samples DNA Extraction, protocol H INRA. INRAE, Jouy-en-Josas, France. http://www.human-microbiome.org/index.php?id=Sop&num=007.

[6] PacBio. Preparing 10-kb library using SMRTbell express template prep kit 2.0 for metagenomics shotgun sequencing. PacBio, Menlo Park, CA. https://www.pacb.com/wp-content/uploads/Procedure-Checklist-%E2%80%93-Preparing-10-kb-Library-Using-SMRTbell-Express-Template-Prep-Kit-2.0-for-Metagenomics-Shotgun-Sequencing.pdf.

[7] PacBio. PacBio CCS. PacBio, Menlo Park, CA. https://github.com/PacificBiosciences/ccs.

[8] Li H. 2018. Minimap2: pairwise alignment for nucleotide sequences. Bioinformatics 34:3094–3100. doi:10.1093/bioinformatics/bty191. 29750242 PMC6137996

[9] Kolmogorov M, Bickhart DM, Behsaz B, Gurevich A, Rayko M, Shin SB, Kuhn K, Yuan J, Polevikov E, Smith TPL, Pevzner PA. 2020. metaFlye: scalable long-read metagenome assembly using repeat graphs. Nat Methods 17:1103–1110. doi:10.1038/s41592-020-00971-x. 33020656 PMC10699202

[10] Kang DD, Li F, Kirton E, Thomas A, Egan R, An H, Wang Z. 2019. MetaBAT 2: an adaptive binning algorithm for robust and efficient genome reconstruction from metagenome assemblies. PeerJ 7:e7359. doi:10.7717/peerj.7359. 31388474 PMC6662567

[11] Pan S, Zhu C, Zhao X-M, Coelho LP. 2022. A deep siamese neural network improves metagenome-assembled genomes in microbiome datasets across different environments. Nat Commun 3:2326. doi:10.1038/s41467-022-29843-y. PMC9051138 35484115

[12] Uritskiy GV, Diruggiero J, Taylor J. 2018. MetaWRAP—a flexible pipeline for genome-resolved metagenomic data analysis. Microbiome 6:158. doi:10.1186/s40168-018-0541-1. 30219103 PMC6138922

[13] Parks DH, Imelfort M, Skennerton CT, Hugenholtz P, Tyson GW. 2015. CheckM: assessing the quality of microbial genomes recovered from isolates, single cells, and metagenomes. Genome Res 25:1043–1055. doi:10.1101/gr.186072.114. 25977477 PMC4484387

[14] Manni M, Berkeley MR, Seppey M, Simão FA, Zdobnov EM. 2021. BUSCO update: novel and streamlined workflows along with broader and deeper phylogenetic coverage for scoring of eukaryotic, prokaryotic, and viral genomes. Mol Biol Evol 38:4647–4654. doi:10.1093/molbev/msab199. 34320186 PMC8476166

[15] Orakov A, Fullam A, Coelho LP, Khedkar S, Szklarczyk D, Mende DR, Schmidt TSB, Bork P. 2021. GUNC: detection of chimerism and contamination in prokaryotic genomes. Genome Biol 22:178. doi:10.1186/s13059-021-02393-0. 34120611 PMC8201837

[16] Seemann T. 2014. Prokka: rapid prokaryotic genome annotation. Bioinformatics 30:2068–2069. doi:10.1093/bioinformatics/btu153. 24642063

[17] Chaumeil P-A, Mussig AJ, Hugenholtz P, Parks DH. 2020. GTDB-Tk: a toolkit to classify genomes with the Genome Taxonomy Database. Bioinformatics 36:1925–1927. doi:10.1093/bioinformatics/btz848. PMC7703759 31730192

[18] Bowers RM, Kyrpides NC, Stepanauskas R, Harmon-Smith M, Doud D, Reddy TBK, Schulz F, Jarett J, Rivers AR, Eloe-Fadrosh EA, Tringe SG, Ivanova NN, Copeland A, Clum A, Becraft ED, Malmstrom RR, Birren B, Podar M, Bork P, Weinstock GM, Garrity GM, Dodsworth JA, Yooseph S, Sutton G, Glöckner FO, Gilbert JA, Nelson WC, Hallam SJ, Jungbluth SP, Ettema TJG, Tighe S, Konstantinidis KT, Liu WT, Baker BJ, Rattei T, Eisen JA, Hedlund B, McMahon KD, Fierer N, Knight R, Finn R, Cochrane G, Karsch-Mizrachi I, Tyson GW, Rinke C, Lapidus A, Meyer F, Yilmaz P, Parks DH, Eren AM, Schriml L, Banfield JF, The Genome Standards Consortium, et al. 2017. Minimum information about a single amplified genome (MISAG) and a metagenome-assembled genome (MIMAG) of bacteria and archaea. Nat Biotechnol 35:725–731. doi:10.1038/nbt.3893. 28787424 PMC6436528

[19] Almeida A, Nayfach S, Boland M, Strozzi F, Beracochea M, Shi ZJ, Pollard KS, Sakharova E, Parks DH, Hugenholtz P, Segata N, Kyrpides NC, Finn RD. 2021. A unified catalog of 204,938 reference genomes from the human gut microbiome. Nat Biotechnol 39:105–114. doi:10.1038/s41587-020-0603-3. 32690973 PMC7801254

[20] Jain C, Rodriguez-R LM, Phillippy AM, Konstantinidis KT, Aluru S. 2018. High throughput ANI analysis of 90K prokaryotic genomes reveals clear species boundaries. Nat Commun 9:5114. doi:10.1038/s41467-018-07641-9. 30504855 PMC6269478

